# Meta-analysis of Fragmented QRS as an Electrocardiographic Predictor for Arrhythmic Events in Patients with Brugada Syndrome

**DOI:** 10.3389/fphys.2017.00678

**Published:** 2017-09-12

**Authors:** Lei Meng, Konstantinos P. Letsas, Adrian Baranchuk, Qingmiao Shao, Gary Tse, Nixiao Zhang, Zhiwei Zhang, Dan Hu, Guangping Li, Tong Liu

**Affiliations:** ^1^Tianjin Key Laboratory of Ionic-Molecular Function of Cardiovascular Disease, Department of Cardiology, Tianjin Institute of Cardiology, Second Hospital of Tianjin Medical University Tianjin, China; ^2^Laboratory of Cardiac Electrophysiology, Second Department of Cardiology, “Evangelismos” General Hospital of Athens Athens, Greece; ^3^Divisions of Cardiology, Queen's University, Kingston General Hospital Kingston, ON, Canada; ^4^Department of Medicine and Therapeutics, Chinese University of Hong Kong Hong Kong, China; ^5^Li Ka Shing Institute of Health Sciences, Chinese University of Hong Kong Hong Kong, China; ^6^Department of Cardiology and Cardiovascular Research Institute, Renmin Hospital of Wuhan University Wuhan, China; ^7^Masonic Medical Research Laboratory Utica, NY, United States

**Keywords:** fragmented QRS, Brugada syndrome, predictor, arrhythmic events, meta-analysis

## Abstract

Fragmented QRS (fQRS) is an electrocardiographic marker related to ventricular fibrillation (VF) and sudden cardiac death (SCD) in various clinical settings. Current data regarding the prognostic significance of fQRS in Brugada syndrome (BrS) are contradictory. This meta-analysis aimed to evaluate the presence of fQRS as a risk stratification tool in BrS. Electronic databases (PubMed, EMBASE, and Cochrane Library) were searched until May 2016. Eight observational studies accumulating data on 1,637 BrS patients (mean age: 47 ± 11 years) were included in this meta-analysis. The mean follow-up duration ranged from 21 to 96 months. fQRS was found to be an independent predictor of future arrhythmic events in BrS (RR:3.88, 95% CI 2.26 to 6.65, *p* < 0.00001) with moderate heterogeneity (I^2^ = 54%, *P* = 0.03). When analyzing VF as independent end-point, the RR for VF was 3.61, and its 95% CI was 2.11 to 6.18, *p* < 0.00001. This meta-analysis showed that BrS patients with fQRS are at high risk for future arrhythmic events. The presence of fQRS warrants prospective evaluation as valid arrhythmogenic risk marker in BrS.

## Introduction

Brugada syndrome (BrS) is a primary arrhythmic syndrome characterized by ST-segment elevation in the right precordial leads on the surface electrocardiogram (ECG) in the absence of overt structural heart disease (Brugada and Brugada, 1992; Mizusawa and Wilde, 2012; Antzelevitch and Patocskai, 2016; Tse et al., 2016). It is associated with a higher risk of ventricular fibrillation (VF) and sudden cardiac death (SCD) compared to the general population. BrS is a genetically heterogeneous ion channelopathy. Up to now, mutations in 19 genes have been identified in subjects with BrS (Mizusawa and Wilde, 2012). This disease typically manifests as cardiac arrest or syncope, occurring in the third and fourth decades of life (Mizusawa and Wilde, 2012; Antzelevitch and Patocskai, 2016). The majority of BrS patients are asymptomatic and diagnoses are often made incidentally. Risk stratification of BrS patients, and particularly those who are asymptomatic, remains challenging (Raju et al., 2011). Fragmented QRS (fQRS) is a simple non-invasive ECG depolarization marker used to identify individuals at high risk of ventricular arrhythmias and SCD in various clinical settings, including coronary artery disease, BrS, long QT syndrome, arrhythmogenic right ventricular cardiomyopathy, and cardiac sarcoidosis (Pietrasik and Zareba, 2012; Jain et al., 2014). It is defined as the presence of an additional R wave (R') or notching in the nadir of the S wave or the presence of >1 R' in two contiguous leads or the presence of more than two notches in the R or S waves in two consecutive leads in the presence of bundle branch block (Jain et al., 2014). Previous studies addressing the prognostic significance of fQRS in subjects with BrS have demonstrated conflicting results (Priori et al., 2012; Take et al., 2012; Tokioka et al., 2014; Calo et al., 2016). Therefore, we performed a comprehensive systematic review and meta-analysis of the current evidence regarding the prognostic significance of fQRS.

## Methods

Two reviewers (L. M. and Q. Z.) independently and systematically performed a literature search on the PubMed, EMBASE, and Cochrane Library databases, to identify relevant studies. We searched the related studies published from May 2006 to 2016 using the following keywords: “Brugada” and “syndrome” or “Brugada syndrome” and “electrocardiography” or “ECG” or “implantable cardioverter defibrillator therapy.” In order to identify all potentially relevant studies, the titles, abstracts, and reference lists of all articles were carefully reviewed. The definition of fQRS included in this meta-analysis, according to previous studies what we synthesized (Das et al., 2007, 2010; Priori et al., 2012), was the presence of an abnormal fragmentation within QRS complex as ≥ two spikes in leads V1 to V3, or the presence of an additional wave or notching in the leads.

Studies were included if they met the following criteria: (a) the study design was a prospective or retrospective observational study; (b) patients with either a spontaneous or a drug-induced type 1 ECG BrS pattern; (c) fQRS was assessed with ECG or Holter monitoring; (d) the follow-up duration was sufficiently long that the arrhythmic events would be detected (the duration of follow-up ≥1 years); (e) endpoint events [appropriate implantable cardioverter-defibrillator therapy (ICD), VF, and SCD] were clearly defined; (f) studies with full-text; and (g) risk ratio (RR), hazard ratio (HR), odds ratio (OR), and the corresponding 95% confidence interval (CI) was reported, or could be calculated. We included both published and unpublished studies without language restriction. In the case of numerous reports by the same group of authors, only the study with the largest number of patients was included. The potential relevant studies were retrieved as full text and assessed for compliance with the inclusion criteria by two investigators. Any uncertainties or discrepancies were resolved through consensus after rechecking the source data and consultation with a third reviewer (T. L.).

Using a standard data extraction form, two blinded reviewers (L. M. and Q. Z.) independently performed data extraction to determine eligibility for inclusion. The extracted data elements of this meta-analysis consisted of: (a) publication details: surname of first author, publication year and location; (b) type of study: multicenter or single center study; (c) study design; (d) follow-up duration; (e) definition of fragmented QRS; (f) endpoint events (arrhythmic events were defined as ventricular fibrillation or flutter, SCD, and the combination of those two during the follow-up period; (g) the quality score; and (h) the characteristics of the population including sample size, gender, age, number of subjects with spontaneous or drug-induced BrS ECG pattern, number of subjects with ICD, number of subjects with family history of SCD or syncope, positive *SCN5A* gene mutation, detailed information in relation to programmed ventricular stimulation (PVS), positive number of inducible VF, and the presence of fQRS.

Quality assessment of these studies included in our meta-analysis was performed using the Newcastle–Ottawa Quality Assessment Scale (NOS; Gussak et al., 2000). The point score system evaluated the categories of study participant selection, comparability of the results, and quality of the outcomes. The following characteristics were assessed: (a) representativeness of the exposed cohort; (b) selection of the non-exposed cohort; (c) ascertainment of exposure; (d) demonstration that outcome of interest was not present at start of study; (e) comparability of cohorts on the basis of the design or analysis; (f) assessment of outcome; (g) follow-up period was sufficiently long for outcomes to be detected; and (h) adequacy of follow up of cohorts. This scale varied from zero to nine stars, which indicated that studies were graded as poor quality if they met <5 criteria, fair if they met 5 to 7 criteria, and good if they met >8 criteria. Studies with a score equal to or higher than six were considered to be concluded.

We extracted and analyzed all the multivariate adjusted RRs, ORs, or HRs with 95% confidence interval (CI) for each study. Pooled effect sizes were presented as the RR with 95% CI for each trial, using the random effects model. Since the related data were occasionally absent, raw data were used to calculate unadjusted risk estimates (Morita et al., 2008; Maury et al., 2013; Tokioka et al., 2014; Calo et al., 2016; Rivard et al., 2016). The HR values in multivariate Cox proportional hazards models in each primary study were directly considered as RR values (Greenland, 1987). To evaluate the heterogeneity across studies, I^2^ derived from the standard chi-square test, which described the percentage of the variability in effect estimates resulting from heterogeneity, was used. I^2^ > 50% is an indicator of significant statistical heterogeneity. In this case, the random-effects model using the inverse variance heterogeneity method was used. Subgroup analyses were performed based on the different primary endpoints and whether pooled effect sizes were adjusted in the patients with BrS. In addition, we performed the sensitivity analysis in a random predefined manner. Publication bias was evaluated using the funnel plot. Statistical significance was defined at *P*-values ≤ 0.05. All analyses were performed using Review Manager, version 5.0.12 (Revman; The Cochrane Collaboration, Oxford, United Kingdom).

## Results

**Figure 1:**
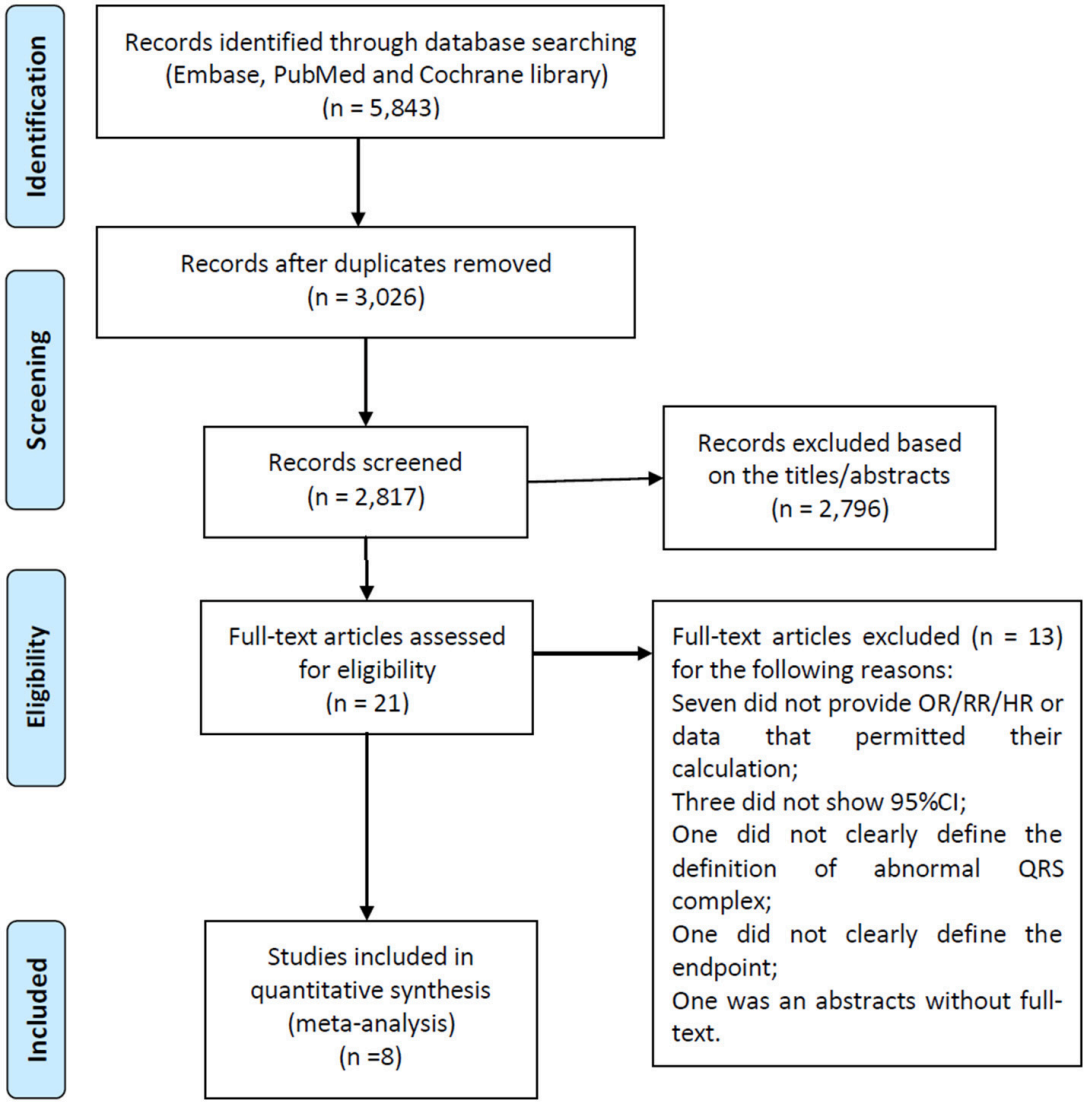
Flow diagram of the study selection process.

A flow diagram of the data search and study selection is presented in Figure 1. A total of 5,843 records were identified using our search criteria. 3,026 duplicate studies were discarded. After screening the titles and abstracts, 2,796 studies were excluded as they were guidelines, editorials, case reports, laboratory studies, animal studies, review articles, or irrelevant to the present study. Therefore, 21 potentially relevant studies were retrieved for detailed evaluation. Of these, 13 were further excluded from the analysis for the following reasons: in 10 studies, RRs, ORs, or HRs were not provided or couldn't be calculated, or 95% confidence intervals were not included; one study did not clearly define the type of the abnormal QRS complex; one failed to clearly define the endpoints, and one was an abstract without full-text.

A total of 1,637 patients (mean age: 47 ± 11 years) with BrS from eight studies (6 prospective cohort studies and 2 retrospective cohort studies) were included in our meta-analysis (Morita et al., 2008; Priori et al., 2012; Take et al., 2012; Maury et al., 2013; Apiyasawat et al., 2014; Tokioka et al., 2014; Calo et al., 2016; Rivard et al., 2016). The baseline characteristics of these studies are listed in Table 1. The mean follow-up duration ranged from 21 to 96 months. The definition of fQRS was similar in all studies. Table 2 shows the patients' characteristics included in each study.

**Table 1:** Study characteristics of eight studies included in meta-analysis.

**Investigator (year)**	**Location**	**Type of study**	**Study design**	**Number of patients(n)**	**Study population**	**Mean follow-up**	**Definition of fQRS**	**Endpoint**	**Quality score**
Morita et al., 2008	Japan	MC	PS	115	Patients with Brugada-type ECG	43 ± 25 months	A^*^	SCD/VF/Non-cardiac death	8
Priori et al., 2012	Italy	MC	PS	308	Patients with type 1 ECGs, without history of cardiac arrest	36 ± 8 months	B^*^	The occurrence of VF or appropriate ICD interventions	9
Take et al., 2012	Japan	SC	PS	84	Patients with a history of syncope or faintness and type 1 ECG	48 ± 48 months	NA	Syncope/VF	8
Maury et al., 2013	France	MC	RS	325	Patients with BrS with spontaneous or drug-induced type 1ECG	48 ± 34 months	NA	Unexplained syncope/malignant ventricular arrhythmias (SCD or ICDth).	6
Apiyasawat et al., 2014	Thailand	SC	PS	107	Patients who underwent an ICD implantation	21.3 ± 23 months	C^*^	Appropriate ICD therapy for ventricular arrhythmia	8
Tokioka et al., 2014	Japan	SC	RS	246	Patients with a Brugada-type ECG	45.1 ± 44.3 months	A^*^	Death/VF or SCD/the last visit	9
Calo et al., 2016	Italy	MC	PS	347	Patients with spontaneous type 1 BrS ECG phenotype	48 ± 38.6 months	D^*^	Syncope/VF/SCD	9
Rivard et al., 2016	Canada	MC	PS	105	Patients with a spontaneous or induced coved type 1 ECG pattern	59.6 ± 16.4 months	A^*^	Aborted SCD or appropriate ICD therapy	9

*BrS, Brugada syndrome; ECG, electrocardiogram; fQRS, fragmented QRS; ICD, implantable cardioverter defibrillator; ICDth, implantable cardioverter defibrillator therapy; MC, multicenter study; NA, not available; n, number; PS, prospective study; RS, retrospective study; SC, single center study; SCD, sudden cardiac death; VF, ventricular fibrillation; VT, ventricular tachycardia*.

A* , an abnormal fragmentation within QRS complex as ≥4 spikes in 1 or ≥8 spikes in all of the leads V1 to V3;

B* , an abnormal fragmentation within QRS complex as ≥2 spikes in leads V1 to V3;

C* , the presence of an additional R wave or notching in the nadir of the S wave in 2 consecutive leads corresponding to a major coronary artery territory or more than 2 notches in the R or S waves in 2 consecutive leads in the presence of bundle branch block;

D* *, an abnormal fragmentation within QRS complex as≥4 spikes in a single lead or ≥8 spikes in leads V1 to V3 as well as evidence of an epsilon wave in the V1 lead*.

**Table 2:** Patients' characteristics of eight included studies.

	**Morita et al., 2008**	**Take et al., 2012**	**Priori et al., 2012**	**Maury et al., 2013**	**Tokioka et al., 2014**	**Apiyasawat et al., 2014**	**Calo et al., 2016**	**Rivard et al., 2016**
Total patients, n	115	84	308	325	246	107	347	105
Male/female, n	113/2	82/2	247/61	258/67	236/10	88/19	272/75	83/22
Age (years)	42 ± 12	47 ± 12	47 ± 12	47 ± 13	47.6 ± 13.6	53	45 ± 13.1	46.2 ± 13.3
Spontaneous Brugada ECG, n (%)	115 (100)	61 (73)	171 (56)	143(44)	156 (63)	NA	347(100)	NA
Drug-induced Brugada ECG, n (%)	0 (0)	23 (27)	137 (44)	182(56)	90 (37)	NA	0 (0)	NA
Patients with ICD, n (%)	40 (35)	45 (54)	137(44)	135(42)	63 (26)	107(100)	98(28.2)	56 (53.3)
Family history of SCD, n (%)	39 (34)	21 (25)	NA	94(29)	69 (28)	NA	71(20.5)	28 (26.7)
History of syncope, n (%)	28 (24.3)	76 (90.4)	65(21)	73 (22)	40(16.3)	NA	14(4.0)	39 (37.1)
SCN5A mutation, n (%)	11 (16.7)	20 (43.5)	24 (20)	42(22)	17 (13.8)	NA	32 (29.9)	13 (20.6)
**PVS**								
Stimulation sites	RVA+RVOT+LV	RVA+ RVOT+LV	RVA+RVOT	NA	RVA+RVOT+LV	NA	RVA+RVOT	RVA+RVOT
Extra stimuli	Up to 2	Up to 3	Up to 3	NA	Up to 3	NA	Up to 2 or 3	up to 3
Basic cycle lengths	2 cycles	2 cycles	600/400/200 ms	NA	2 cycles	NA	2 cycles	400 and 600 ms
Patients with PVS, n (%)	NA	72(86)	308 (100)	219 (67)	246 (100)	35(32.7)	77 (41.4)	56 (53.3)
Inducible VT/VF, n (%)	NA	38 (45)	126 (41)	93(42)	71 (29)	35(32.7)	65 (18.7)	23 (41.1)
fQRS (+), n (%)	50(43)	37 (44)	25(8.1)	8(2.5)	78(31.7)	42(39.3)	85 (24.5)	6 (7.5)
Endpoint in group with fQRS (+), n(N)	17(50)	23(37)	7(25)	2(8)	20(78)	14(42)	11(85)	2(6)
Endpoint in group with fQRS (−), n(N)	1(65)	9(47)	7(283)	24(317)	4(168)	6(65)	21(262)	14(99)

*Data were presented as mean ± SD, median, or percentage where possible; ECG, electrocardiogram; fQRS, fragmented QRS; ICD, implantable cardioverter defibrillator; LV, left ventricle; NA, not available; n, number; PVS, programmed ventricular stimulation; RVA, right ventricular apex; RVOT, right ventricular outflow tract; SCD, sudden cardiac death; VT, ventricular tachycardia; VF, ventricular fibrillation*.

**Figure 2:**
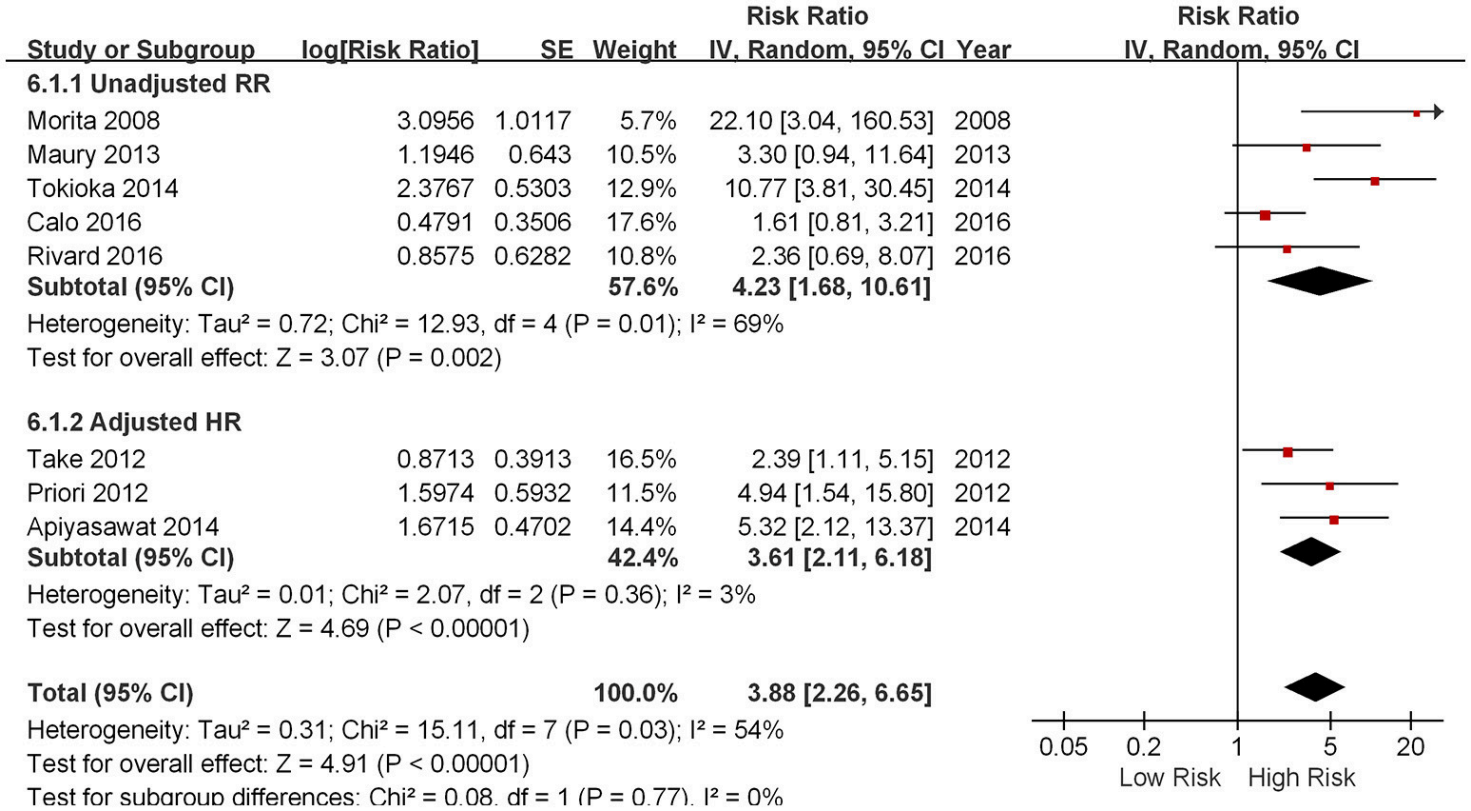
Forest plot demonstrating the association between fQRS and the fatal arrhythmias events and sudden cardiac death in the patients with Brugada syndrome.

Five out of eight studies demonstrated that the presence of fQRS predicted future arrhythmic events in patients with a spontaneous or drug-induced type 1 ECG pattern of BrS (Morita et al., 2008; Priori et al., 2012; Take et al., 2012; Apiyasawat et al., 2014; Tokioka et al., 2014). On the contrary, three studied failed to show any prognostic significance of fQRS (Maury et al., 2013; Calo et al., 2016; Rivard et al., 2016). As shown in Figure 2, the pooled meta-analysis of eight included studies demonstrated that the presence of fQRS is an independent predictor of future arrhythmic events in BrS (RR: 3.88, 95% CI 2.26 to 6.65, *p* < 0.00001) with moderate heterogeneity (I^2^ = 54%, *P* = 0.03).

Subgroup analysis was performed based on different arrhythmic endpoints, When analyzing studies using VF/SCD as a combined endpoint, pooled analysis of four studies showed that fQRS was an independent predictor of future arrhythmic events (RR 3.36, 95% CI 2.03 to 5.56, *p* < 0.00001) with moderate heterogeneity (*p* = 0.005 and I^2^ = 76%; Morita et al., 2008; Maury et al., 2013; Tokioka et al., 2014; Calo et al., 2016). When analyzing VF and SCD as different end-points, the RRs were the following: VF from three studies (RR 3.61, 95% CI 2.11 to 6.18, *p* < 0.00001; Priori et al., 2012; Take et al., 2012; Apiyasawat et al., 2014) and SCD in one study (RR 2.36, 95% CI 0.69 to 8.07, *p* = 0.9502; Rivard et al., 2016).

We also performed subgroup analysis based on whether pooled effect sizes was adjusted from each study. Regardless of whether adjustment of potential confounding variables were made, the outcomes were similar: unadjusted RR (4.23, 95% CI 1.68 to 10.61, *p* = 0.002; Morita et al., 2008; Maury et al., 2013; Tokioka et al., 2014; Calo et al., 2016; Rivard et al., 2016) and adjusted HR (3.61, 95% CI 2.11 to 6.18, *p* < 0.00001; Priori et al., 2012; Take et al., 2012; Apiyasawat et al., 2014).

**Figure 3:**
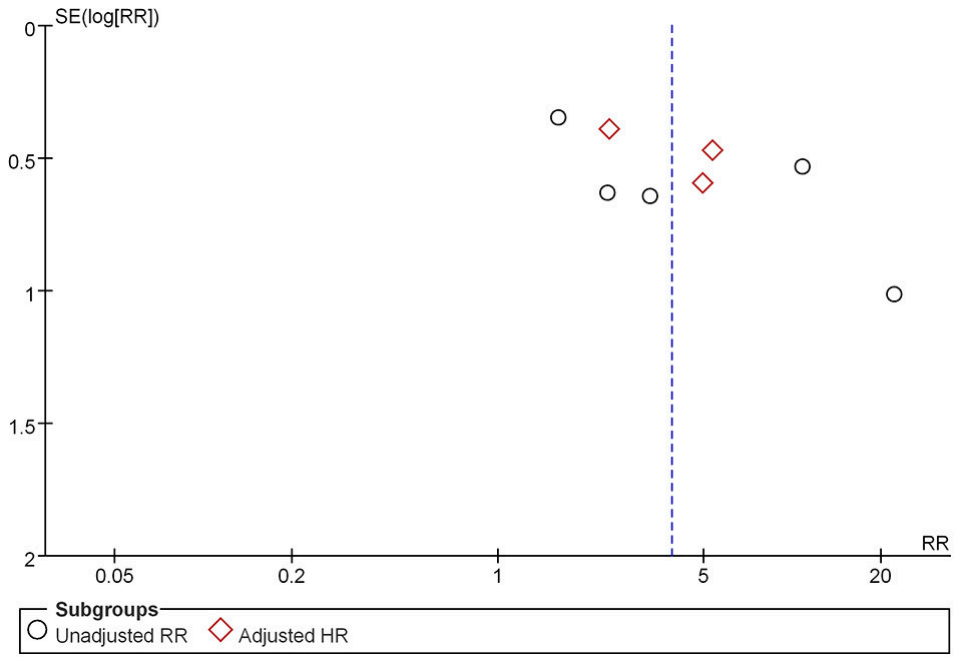
Funnel plot of the meta-analysis.

Sensitivity analysis was then performed to identify possible causes of the significant heterogeneity in our meta-analysis. After excluding the studies which the RR value was calculated according to the raw data, no significant heterogeneity was found in the remaining studies (I^2^ = 3%). As a consequence, a potential source of the heterogeneity observed in our meta-analysis may be related to the different measures used for risk assessment. As shown in the Figure 3, the results of the funnel plot suggested that little publication bias was present.

## Discussion

The main findings of the present meta-analysis are that: (1) overall, the presence of fQRS is associated with a 3-fold increased risk of future arrhythmic events in patients with BrS, and (2) the presence of fQRS confers a 3- and 2-fold increase for VF and SCD events, respectively. We further performed the subgroup and sensitivity analyses to identify possible causes of the significant heterogeneity in the analysed studies. The results showed that the calculation method of risk ratios is a possible origin of heterogeneity observed in our meta-analysis.

Asymptomatic BrS patients display an annual event rate of arrhythmic events between 0.5 and 1% (Priori et al., 2012). Such arrhythmic events occur in about 50% of cases as VF without prior warning symptoms (Jain et al., 2014). Risk stratification of BrS patients is therefore of paramount importance. Several ECG markers have been proposed for risk stratification of BrS patients (Probst et al., 2010; Tse, 2016; Tse and Yan, 2016a,b; Tse et al., 2017), but the majority have not been tested in a prospective manner (Mizusawa and Wilde, 2012).

fQRS is a relatively new arrhythmogenic ECG marker validated in different clinical settings (Raju et al., 2011; Pietrasik and Zareba, 2012). In a recent meta-analysis of 5,009 patients with coronary artery disease and non-ischemic cardiomyopathy, the presence of fQRS was associated with a relative risk for SCD of 2.2 (Rosengarten et al., 2015). Data regarding the prognostic significance of fQRS in BrS are limited. Morita et al. have demonstrated that fQRS is more commonly seen in BrS patients with VF (85%) and syncope (50%) compared to asymptomatic ones (34%; Morita et al., 2008). The PRELUDE study confirmed these findings and showed that fQRS is an independent predictor of future arrhythmic events (HR: 4.94; Priori et al., 2012). Tokioka et al. recently demonstrated that the presence of QRS-fragmentation lead to a 5-fold increase in the incidence of arrhythmic events (VF, SCD; Tokioka et al., 2014). ICD is the golden therapeutic method for cardiac arrest. The study by Apiyasawat et al. had confirmed that fQRS was directly associated with appropriate ICD therapy (Apiyasawat et al., 2014). However, the choice of an appropriate ICD intervention as surrogate endpoint for SCD event may lead to overestimation of the potential risk.

The presence of fQRS on surface ECG has been attributed to scar-related inhomogeneous conduction of action potentials through the ventricles. The different morphologies of fQRS are caused by shifting of the QRS vector in and around the fibrotic areas during depolarization, depending on their extent and location in the ventricles (Jain et al., 2014). The pathophysiology of BrS is only partially resolved. So far, repolarization, depolarization, and the current mismatch hypotheses are thought to underlie the development of VF in BrS (Nishii et al., 2010). There is increasing evidence suggesting that mild structural abnormalities observed in right ventricular outflow tract provide the arrhythmia substrate in BrS. Thus, BrS has been recently associated with increased collagen and fibrosis, and reduced gap junction expression in the right ventricular outflow tract. It is therefore quite possible that fQRS in BrS reflects conduction delay in the right ventricle. Indeed, using an isolated canine right ventricular tissue model of BrS, Morita et al. demonstrated that activation delay in the epicardium reproduces fQRS in the transmural ECG (Morita et al., 2008).

## Study limitations

Our study has several potential limitations. Firstly, the small size of the study population may have an important impact in our findings. Second, a potential publication bias was identified in the funnel plot. Thirdly, some studies included in our studies are retrospective studies, which may more recall bias. Fourthly, a potential overlap exists in the studies by Take et al. (2012) and Tokioka et al. (2014). Fifthly, the control variables used for adjusted and unadjusted RR/HR in the different studies may influence the final results of our meta-analysis. However, the subgroup analysis based on whether risk ratio was adjusted from the potential confounding variables had similar final outcomes. Finally, the different measures used for risk assessment may have contributed to the heterogeneity in this meta-analysis.

## Conclusion

In conclusion, the presence of fQRS is predictive of future arrhythmic events in patients with BrS. The presence of fQRS warrants prospective evaluation and validation as a risk marker for clinical use in BrS.

## Author contributions

LM and KL participated in study design, searched databases, extracted and assessed data, and drafted the manuscript. AB, GT, DH, and GL revised the manuscript, QS, NZ, and ZZ participated in extracted data, TL designed the study and revised the manuscript. All authors approved the final version of the manuscript.

### Conflict of interest statement

The authors declare that the research was conducted in the absence of any commercial or financial relationships that could be construed as a potential conflict of interest.
